# Simulation of high-energy radiation belt electron fluxes using NARMAX-VERB coupled codes

**DOI:** 10.1002/2014JA020238

**Published:** 2014-10-06

**Authors:** I P Pakhotin, A Y Drozdov, Y Y Shprits, R J Boynton, D A Subbotin, M A Balikhin

**Affiliations:** 1 Department of Automatic Control and Systems Engineering, University of SheffieldSheffield, UK; 2 Department of Earth and Space Sciences, University of CaliforniaLos Angeles, California, USA; 3 Skobeltsyn Institute of Nuclear Physics, Lomonosov Moscow State UniversityMoscow, Russia; 4 Department of Atmospheric and Planetary Sciences, Massachusetts Institute of TechnologyCambridge, Massachusetts, USA; 5 Skolkovo Institute of Science and TechnologySkolkovo, Russia

**Keywords:** radiation belts, space weather

## Abstract

This study presents a fusion of data-driven and physics-driven methodologies of energetic electron flux forecasting in the outer radiation belt. Data-driven NARMAX (Nonlinear AutoRegressive Moving Averages with eXogenous inputs) model predictions for geosynchronous orbit fluxes have been used as an outer boundary condition to drive the physics-based Versatile Electron Radiation Belt (VERB) code, to simulate energetic electron fluxes in the outer radiation belt environment. The coupled system has been tested for three extended time periods totalling several weeks of observations. The time periods involved periods of quiet, moderate, and strong geomagnetic activity and captured a range of dynamics typical of the radiation belts. The model has successfully simulated energetic electron fluxes for various magnetospheric conditions. Physical mechanisms that may be responsible for the discrepancies between the model results and observations are discussed.

## 1. Introduction

The Van Allen radiation belts are composed of energetic charged particles that are trapped by the Earth's magnetic field. The electron belts exhibit a two-zone structure, with the inner and outer belts separated by an area of reduced flux known as the slot region. The relatively stable inner belt is located below 2 Earth radii, while the highly dynamic electrons of the outer belt can be found at distances greater than ≈ 3 *R*_*E*_. These energetic particles can affect geostationary satellites and damage spacecraft electronics [*Baker*, 1996]. As such, much attention has been devoted to developing accurate space weather prediction models to mitigate hazards caused by varying geomagnetic conditions.

Particles trapped in the radiation belts undergo periodic motion described by adiabatic invariants. Interactions that occur on timescales faster than the periodic motion in question lead to violations of the corresponding adiabatic invariant and to diffusion of the particle in energy or pitch angle. This can happen through wave-particle interactions, and the effect of these violations on the bulk particle distribution is described by quasi-linear theory  [*Vedenov et al.*, 1961; *Kennel and Engelmann*, 1966].

*Friedel et al.* [2002] identified several mechanisms for the buildup of relativistic electrons in radiation belts. The most significant ones are believed to be radial diffusion and local acceleration [e.g., *Horne and Thorne*, 1998; *Summers et al.*, 1998; *Elkington et al.*, 1999; *Green and Kivelson*, 2004]. Radial diffusion is caused by gradients in the phase space density (PSD) of particles and can be enhanced by ultralow frequency (ULF) waves violating the third adiabatic invariant. Particles will diffuse radially inward or outward depending on the sign of the PSD gradient.

Local acceleration is caused by resonant wave-particle interactions with collective plasma instabilities found in the magnetosphere. These can change a particle's pitch angle and energy via violation of the first and second invariants. In particular, energy diffusion by chorus waves creates a peak in phase space density at L ≈ 4 [e.g., *Summers et al.*, 1998; *Brautigam and Albert*, 2000; *O'Brien et al.*, 2003; *Chen et al.*, 2006; *Iles et al.*, 2006; *Shprits et al.*, 2007; *Xiao et al.*, 2009a,^2010^; *Reeves et al.*, 2013; *Thorne et al.*, 2013a; *Xiao et al.*, 2014]. It is postulated that the accelerated electrons can then be transported via radial diffusion to geostationary orbit, providing the bulk of high-energy (>2 MeV) particles encountered at these radial distances [e.g., *Varotsou et al.*, 2005; *Shprits et al.*, 2009]. Meanwhile, chorus, hiss, and electromagnetic ion cyclotron (EMIC) waves contribute to pitch angle scattering of trapped particles and their demise from the system via the loss cone [*Xiao et al.*, 2009b,^2011^, ^2012^]. Another loss process is magnetopause shadowing [e.g., *Desorgher et al.*, 2000], where drifting particles come into contact with the magnetopause on the dayside and escape to interplanetary space. The loss to the magnetopause can cause outward radial diffusion and extend losses down to the heart of the outer belt (below L ≈ 4) [*Shprits et al.*, 2006a; *Turner et al.*, 2012].

The physics behind radiation belt storm dynamics are not yet fully understood, and physics-based models, in particular, the wave models, are in a constant state of development. Additionally, they need a measure of the electron population entering the inner magnetosphere from the tail [e.g., *Ganushkina et al.*, 2013, 2014]. Such a quantity is quite hard to measure empirically. Therefore, at present some of the most accurate models of radiation belt flux forecasting are data driven [e.g., *Baker et al.*, 1990; *Li et al.*, 2001; *Kellerman et al.*, 2013]. In particular, the NARMAX (Nonlinear AutoRegressive Moving Averages with eXogenous inputs) model uses system identification to deduce a mathematical model from recorded data sets by identifying important model terms [*Boynton et al.*, 2013a]. In that sense it is similar to the nonlinear equivalent of a correlation function. The input data is integral high-energy electron fluxes at geostationary orbit using data from the GOES-13 satellite, as well as solar wind parameters from L1. However, data-driven models need continuous data to generate predictions, and the NARMAX approach using geosynchronous orbit (GSO) data gives no indication of what happens at different radial distances.

This study presents a system that merges the physical and data-driven approaches, combining the accuracy of the data-driven NARMAX algorithm with the predictive range and resolution of the physics-based VERB code [*Shprits et al.*, 2008a; *Subbotin and Shprits*, 2009; *Shprits et al.*, 2009]. The VERB code is used to produce an electron phase space density profile as a function of energy, pitch angle, time, and L^*^, where L^*^ refers to the dimensionless Roederer L^*^ [*Roederer*, 1970] and is related to the third adiabatic invariant through


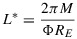
(1)

where *M* is the Earth's dipole magnetic moment, *Φ* is magnetic flux enclosed by the guiding electron drift shell, and *R*_*E*_ is radius of Earth. The VERB code takes two inputs: the geomagnetic *Kp* index and a measure of electrons entering the inner magnetosphere from the tail. While the *Kp* index is in public access and can be forecast with existing models, seed electron fluxes can be quite difficult to estimate. *Subbotin et al.* [2011a] used outputs from the Rice Convection Model (RCM) [*Toffoletto et al.*, 2003 and references therein] as an input to the outer boundary. Combining the data-driven NARMAX model with the VERB code is an alternate solution and is employed in this study. The resulting scheme is called 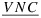
—VERB-NARMAX Coupling—and generates accurate predictions of the entire outer belt region for a range of magnetospheric conditions.

The rest of the paper is split into several sections. Section 2 summarizes the satellite instrumentation and orbital parameters, section 3 provides detailed descriptions of both models, and the coupling method is explained in section 4. Section 5 presents the results and section 6 summarizes the conclusions and caveats and outlines future work.

## 2. Satellite Instrumentation

GOES-13 was launched into geostationary orbit at 75° west longitude. Analysis in this study was performed with GOES-13 data, because these are the data that drive the NARMAX model. It is useful to note that GOES-13 reaches magnetic midnight at 0500 UT and at magnetic noon at 1700 UT. The spacecraft carries particle detectors that measure integral flux of > 800 keV, > 2 MeV, and > 4 MeV electrons, as well as protons of up to >100 keV, in units of particles/(cm^2*^sec^*^sr). It is data from these detectors that drive the NARMAX model. The > 800 keV electron fluxes are used in this study for validation.

The spacecraft pass through different regions in L^*^ at different magnetic local times. Although their orbital diameters remain constant, due to the asymmetry of the magnetic field the spacecraft enter regions of higher magnetic flux intensity on the dayside and lower magnetic flux intensity at magnetic nightside. Since magnetic flux and L^*^ are inversely related, GOES-13 will find itself at the highest L^*^ around 0500 UT and lowest L^*^ at 1700 UT. Particle flux is inversely related to L^*^ because the heart of the radiation belt is inward of the geostationary orbit, so during noon at low L^*^ values, particle fluxes tend to maximize, while at magnetic midnight they drop to minimum levels.

The Van Allen probes were launched in 2012 into near-equatorial orbits that cover the entire radiation belt region, reaching almost to geosynchronous orbit. The MagEIS instruments [*Blake et al.*, 2013] aboard the twin probes measure electron flux over a wide range of energy channels. Assuming an exponential spectrum of particle fluxes, it is possible to directly compare MagEIS measurements with the simulation. Data from the RBSP-B probe were used in this study.

## 3. Model Description

### 3.1 Electron Flux Forecasting With the NARMAX Model

NARMAX (or nonlinear autoregressive moving average model with exogenous inputs) is a data-driven methodology [*Billings et al.*, 1989] that has been used to study nonlinear dependencies of the dynamics of the magnetosphere [e.g., *Boaghe et al.*, 2001; *Balikhin et al.*, 2001,2010; *Boynton et al.*, 2011,2013b]. In this scheme the output at time *t* is a scalar value that is assumed to be a function of previous values of inputs *u*(*t*), output *y*(*t*), and error terms *e*(*t*) as described by the following equation:


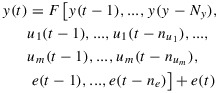
(2)

where *F*[·] is some nonlinear function, *y*,*u*, and *e* are output, input, and error terms, *m* is the number of inputs to the system, and *n*_*y*_,*n*_*u*1_,...,*n*_*um*_, and *n*_*e*_ are the maximum time lags of the output, the *m* inputs and error, respectively.

In this study, NARMAX is used to predict integral electron fluxes for the next 24 h given data from two GOES-13 satellite channels for detecting > 800 keV and > 2 MeV and solar wind parameters from L1. The output is in the form of daily predictions of fluxes of > 800 keV and > 2 MeV electrons in units of *n*_*e*_/d/cm^2^/sr.

### 3.2 Radiation Belt Simulation With the VERB Code

The Versatile Electron Radiation Belt (VERB) code [e.g., *Subbotin et al.*, 2011b, and references therein] is a radiation belt simulation code that models radiation belt particle dynamics as a diffusion equation [e.g., *Schulz and Lanzerotti*, 1974; *Shprits et al.*, 2008b; *Albert et al.*, 2009] with diffusion in radial distance, pitch angle, energy, and mixed terms:


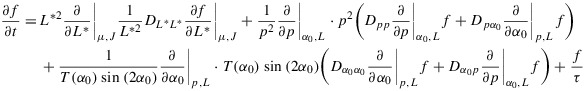
(3)

here, *f* is the electron phase space density, *t* is time, *p* is relativistic momentum, *α*_0_ is equatorial pitch angle, *L* is McIlwain's magnetic shell parameter (radial distance from the center of the Earth to the equatorial field line point) and L^*^ is the Roederer parameter.

The *μ*, *J*, and *Φ* are the three adiabatic invariants that may be expressed as


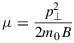
(4)


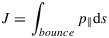
(5)


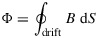
(6)

*T*(*α*_*p*_) in (1) is a function related to the bounce frequency and is approximated by *Lenchek et al.* [1961]


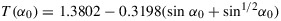
7

Radial diffusion is represented by the first term on the right-hand side of the equation, while diffusion in energy (momentum), pitch angle, and mixed terms are represented by the next four. Losses to the atmosphere are represented by the last term *f*/*τ* where *τ* is a characteristic electron lifetime assumed to be infinite outside the loss cone and equal to a quarter bounce time inside it. All pitch angle and energy diffusion coefficients are parameterized by *Kp* index either explicitly or implicitly through plasmapause location. They are bounce- and MLT-averaged and data on wave-particle interactions are drawn from statistical studies.

The first term in (1) is the radial diffusion term written in terms of *μ*, *J*, and L^*^ and describes the radial diffusion of PSD with the radial diffusion coefficient 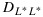. Radial diffusion violates the third invariant while conserving the first and second. Meanwhile, wave-particle interactions violate the first and second invariants, leading to diffusion in energy and pitch angle. Radial diffusion is assumed to be driven by resonant interactions with ULF waves, with diffusion coefficient due to the magnetic component of ULF adopted from *Brautigam and Albert* [2000]:


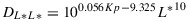
(8)

The radial diffusion coefficient was parameterized for the *Kp* index below 6, however, in the VERB model the same parameterization is used for all *Kp* indices.

The last four terms describe diffusion in momentum (*D*_*pp*_), pitch angle 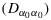, and mixed diffusion terms 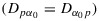. Energy and relativistic momentum are related via 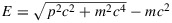 where *c* is the speed of light.

The waves used in the simulation are dayside chorus, nightside chorus, and plasmaspheric hiss. The assumed wave model for the chorus waves is reproduced on Table 1 from  *Subbotin et al.* [2011b]. Plasmaspheric hiss data are used from *Orlova et al.* [2014] where the wave normal angle distribution was taken from Table S3 in the supporting information of  *Thorne et al.* [2013b]. The plasmapause location is calculated using *Carpenter and Anderson* [1992]:


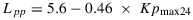
(9)

where *Kp*_max24_ is the maximum *Kp* values in the preceding 24 h. The diffusion coefficients are parameterized by the *Kp* index. VERB assumes a dipole field geometry for the computation and bounce averaging of pitch angle and energy diffusion coefficients.

**Table 1:** Wave Parameters Used for the Diffusion Coefficients Computation

Type of Wave	*B*_*W*_ (pT)	*λ*_max_	Density Model	Percent MLT	Wave Spectral Properties	Distribution in Wave Normal
Chorus day	10^0.75 + 0.04*λ*^(2 × 10^0.73 + 0.91*Kp*^)^0.5^/	35	*Sheeley et al.* [2001]	25%	*ω*_*m*_/*Ω*_*e*_ = 0.2,	*θ*_*m*_ = 0°, *δθ* = 30°,
	57.6 for *Kp*≤ 2+;				*δω*/*Ω*_*e*_ = 0.1,	*θ*_*uc*_ = 45°, *θ*_*lc*_ = 0°.
	10^0.75 + 0.04*λ*^(2 × 10^2.5 + 0.18*Kp*^)^0.5^/				*ω*_*uc*_/*Ω*_*e*_ = 0.3,	
	57.6 for 2 + <*Kp*≤ 6;				*ω*_*lc*_/*Ω*_*e*_ = 0.1,
Chorus night	50(2 × 10^0.73 + 0.91*Kp*^)^0.5^/	15	*Sheeley et al.* [2001]	25%	*ω*_*m*_/*Ω*_*e*_ = 0.35,	*θ*_*m*_ = 0°, *δθ* = 30°,
	57.6 for *Kp*≤ 2+;					*θ*_*uc*_ = 45°, *θ*_*lc*_ = 0°.
	50(2 × 10^2.5 + 0.18*Kp*^)^0.5^/				*ω*_*uc*_/*Ω*_*e*_ = 0.65,	
	57.6 for 2+ <*Kp*≤ 6;				*ω*_*lc*_/*Ω*_*e*_ = 0.05,	*δω*/*Ω*_*e*_ = 0.15,
Plasmaspheric hiss	[*Orlova et al.*, 2014]	*Denton et al.* [2004,2006]	45	62.5%	*f*_*m*_ = 550 Hz, *δf* = 300 Hz,	*Thorne et al.* [2013b]
					*f*_*lc*_=100 Hz, *f*_*uc*_ = 2000 Hz	
					*f*_*uc*_= 2000 Hz, *f*_*lc*_ = 100 Hz,

VERB solves equation 1 numerically for L^*^ from 1 to 7 where 7 is assumed to be the outer simulation boundary. An implicit finite differences method is used on an orthogonal grid for radial, energy, and equatorial pitch angle diffusion. Six boundary conditions are specified, two for each variable in the equation. The two pitch angle boundary conditions are as follows: zero PSD gradient at 0° and 90°. The PSD for low-energy electrons is constant to represent a balance of convective source and losses, while the PSD for high-energy electrons is zero (very few electrons are found at very high energies). Finally, there are assumed to be no electrons present at the low L^*^ boundary to represent atmospheric losses. The high L^*^ boundary is time dependent and is based on CRRES data. Following *Brautigam and Albert* [2000], an energy-independent flux *B*_*f*_(*t*) is used to reproduce the variation in convected outer boundary electrons. It is this parameter that requires NARMAX. Fluxes predicted by NARMAX at GSO are translated into expected differential fluxes at the outer boundary using a procedure outlined in the next section. These are translated from differential fluxes in standard units to fluxes as a function of *B*_*f*_(*t*) and input into the VERB code. This, along with the *Kp* index, provides all the necessary inputs to run the simulation.

## 4. Coupled Simulation

The VERB code was initialized with *Kp* index values from the German Research Centre for Geosciences Web site (http://www-app3.gfz-potsdam.de/kp_index/). The boundary flux— the second input to the system—was taken from NARMAX predictions for fluxes of > 800 keV and > 2 MeV. Differential flux was calculated from these two integral channels assuming exponential energy distributions for an energy of 1 MeV. The reason for this choice was to avoid extraneous dynamics that begin to predominate at higher energies [*Shprits et al.*, 2013]. Because NARMAX predicts for geostationary orbit, it was necessary to recalculate fluxes for the outer boundary at L^*^ = 7. A representative value of the average L^*^ location of geostationary orbit was taken at L^*^ = 6.2. Fluxes were recalculated using the assumptions that PSD remains constant at fixed first invariant between L^*^ = 6.2 and L^*^ = 7. The recalculated fluxes formed the outer boundary condition for VERB. A profile of particle flux as a function of time and L^*^ was generated for an energy of 892 keV so as to compare it directly to output from the Van Allen probes mission MagEIS particle detector channel that has the same energy. This particular channel was chosen because it registers relatively high-energy particles that are nevertheless below 1 MeV.

From the profile generated by VERB, the predicted flux was extracted for each hour at geostationary orbit. Geostationary orbit location in L^*^ is calculated using the ONERA library and the *Kp*-driven T89 magnetic field model [*Tsyganenko*, 1989]. The differential flux taken from the model at 892 keV and 1000 keV was used to calculate flux from the integral energy channel > 800 keV to compare with GOES-13 data.

## 5. Results

Electron flux observations from the GOES-13 and Van Allen probe spacecraft of electron energies above 800 keV were compared with fluxes predicted by the coupled model. This was done for several time periods. The first event, from 1 to 25 August 2013, featured quiet periods punctuated by extended periods of relatively high geomagnetic activity with storm sudden commencements (SSCs). The second time period, 1 to 23 September 2013, is largely geomagnetically quiet with *Kp* index never rising above 3+. The third time period, 1 to 20 October 2013, featured very rapid changes of *Kp* indices and instances of high geomagnetic activity.

### 5.1 First Period: 1–25 August 2013

**Figure 1:**
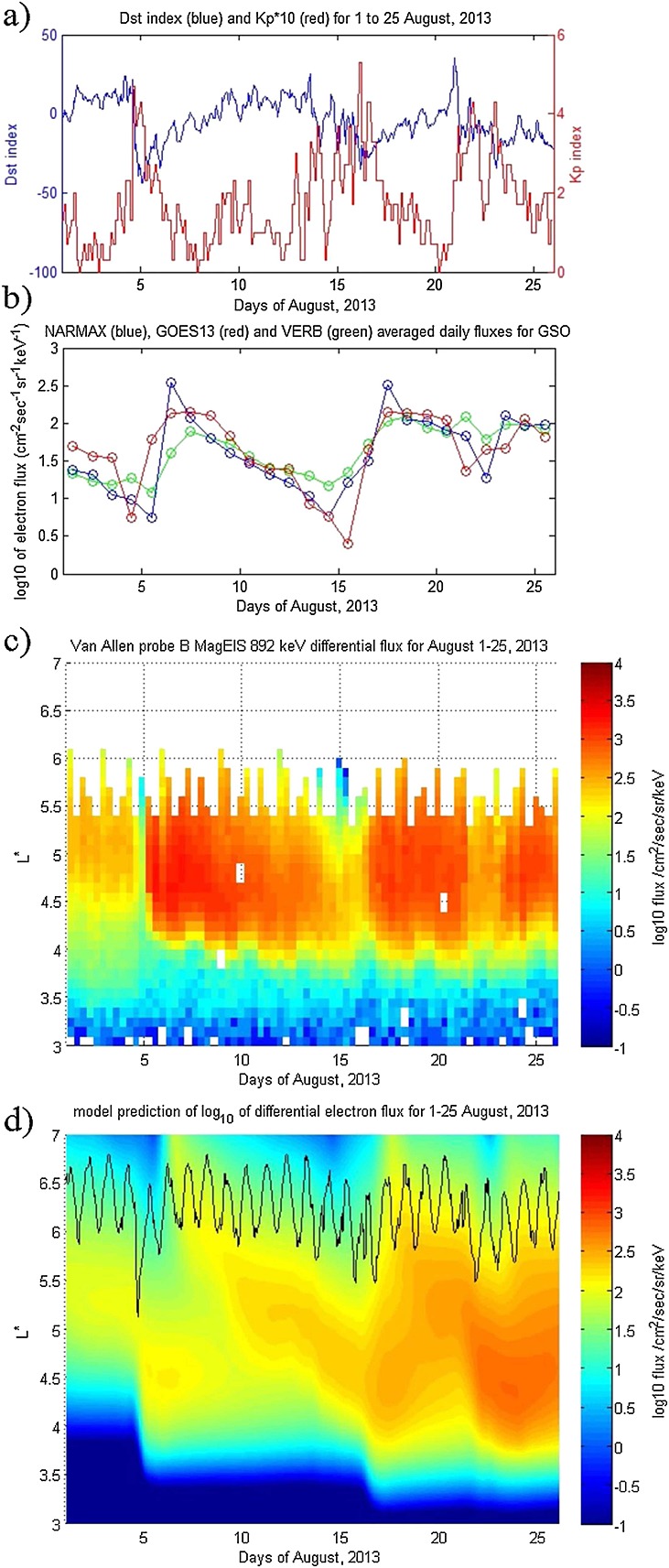
For 1–25 August 2013, (a) *Dst* and *Kp* indices, (b) differential flux for NARMAX, GOES-13, and VERB, (c) Van Allen probe B MagEIS data for 892 keV, and (d) VERB simulation of the outer radiation belt using the NARMAX-derived differential flux as the outer boundary. The black curve in Figure 1d represents geostationary orbit.

Figure 1 shows, for the time period 1–25 August 2013 (a) the *Dst* and *Kp* indices, (b) the differential daily averaged flux from GOES-13, NARMAX, and VNC at geostationary orbit, (c) data from the RBSP-B probe for an energy channel corresponding to 892 keV, and (d) the VERB simulation with a NARMAX-driven outer boundary. It can be seen that there are three periods of electron flux enhancements: 1–4 August, 5–16 August, and 17–26 August with a small decrease in flux around 22–23 August. All these are reproduced by NARMAX (Figure 1b) and as a consequence reflect conditions on the outer boundary in the VNC simulation (Figure 1d). The intensity of dropouts is somewhat underestimated by the model, while fluxes on 4 August exceeded NARMAX predictions and, consequently, VNC reported smaller fluxes than were measured by the Van Allen probe. Otherwise, the model successfully captured the dynamics of this time period, with all three periods of activity visible on Figure 1d.

The reason for the underestimation of fluxes on 4 August can be deduced from observing the input to the outer boundary (Figure 1b). On 5 August, increased flux was observed on GOES-13 (red) but not NARMAX (blue) which in this case registered the increase with a delay. The injection can be seen on the model (Figure 1d) around 6 August; the *Kp* index drops at this point. Since acceleration wave models in VERB are parameterized by the *Kp* index, the magnitude of these processes was too low to reproduce the quite intense flux profile seen on MagEIS data for that time. Thus the variation in this case is a consequence of the fact that VNC is driven not by real GOES-13 data but by the NARMAX predictions of it.

The GOES-13 geosynchronous satellite orbit is represented on Figure 1d as a black sinusoidal curve between L^*^=5 − 7. The location of GSO in L^*^ space varies throughout the day.

### 5.2 Second Period: 1–23 September 2013

**Figure 2:**
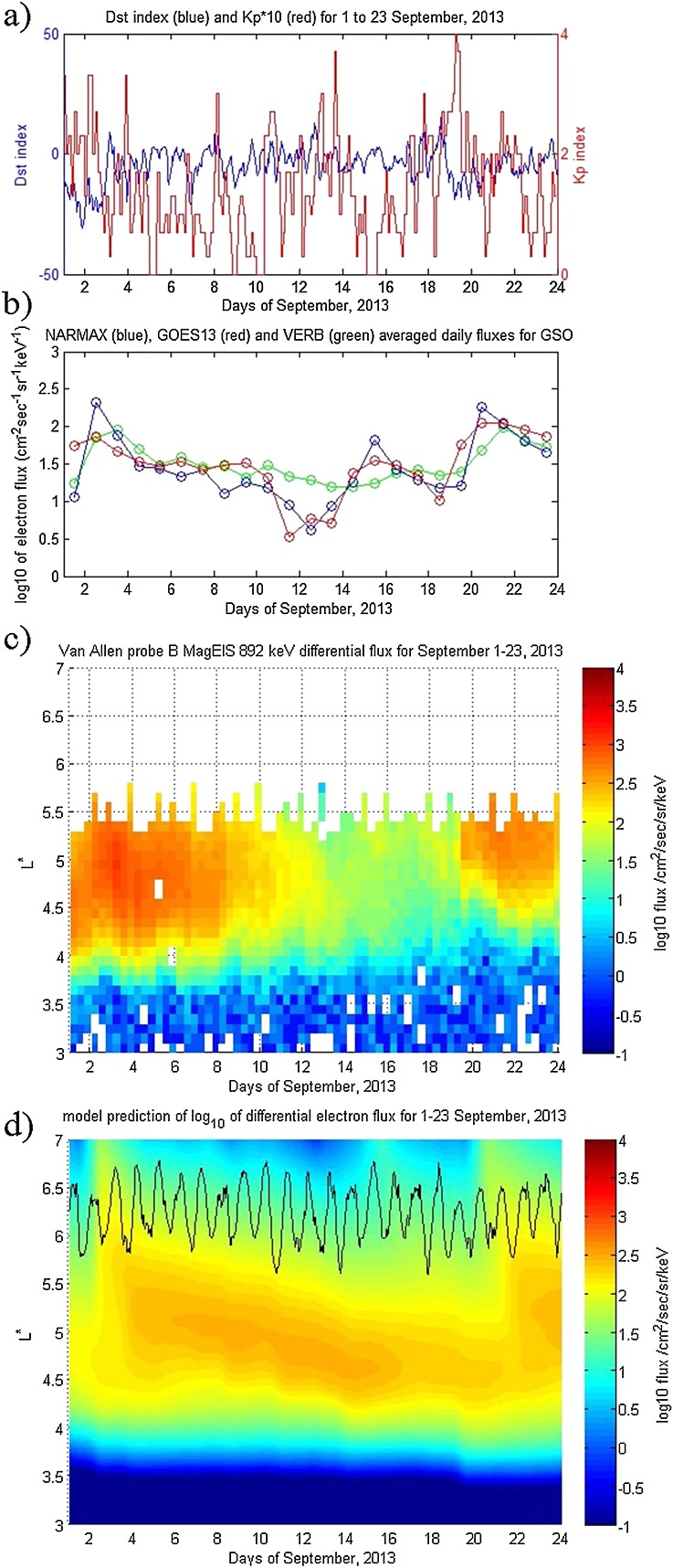
For 1–23 September 2013, (a) *Dst* and *Kp* indices, (b) differential flux for NARMAX, GOES-13, and VERB, (c) Van Allen probe B MagEIS data for 892 keV, and (d) VERB simulation of the outer radiation belt using the NARMAX-derived differential flux as the outer boundary. The black curve in Figure 2d represents geostationary orbit.

Figure 2 shows the same information as Figure 1 for the time period 1–23 September 2013. It can be seen here that under these somewhat more quiet conditions, energetic electron fluxes are lower and have two main enhancements, 1–11 September and 19–24 September. In particular, the time periods 1–11 September features a long, steady period of high fluxes around L^*^ = 5, which subsequently falls off by 13 September. There is another small enhancement barely visible on Figure 2c on 15–16 September on the edge of the satellite's orbital coverage. It can be seen more clearly in Figure 2b from both GOES and NARMAX, suggesting that the particles came from the outer boundary through geostationary orbit. The long buildup of electron flux can be seen on Figure 2d with electron loss again underestimated. The drop in fluxes between 13 and 19 September could have been caused by magnetopause shadowing or by enhanced EMIC activity. The second flux increase on 19 September is successfully simulated by the VNC model.

This period clearly showcases what happens when the VNC scheme is run in quiet geomagnetic conditions. Without any enhanced loss processes, fluxes in the local acceleration zone remain there. This may cause an over-estimation of fluxes in that area over time. Nevertheless, on Figure 2b, flux drop is evident at L^*^=4 − 5 between 14 and 20 September, despite low *Kp* indices and an aforementioned small injection on 15-16 September.

### 5.3 Third Period: 1–20 October 2013

**Figure 3:**
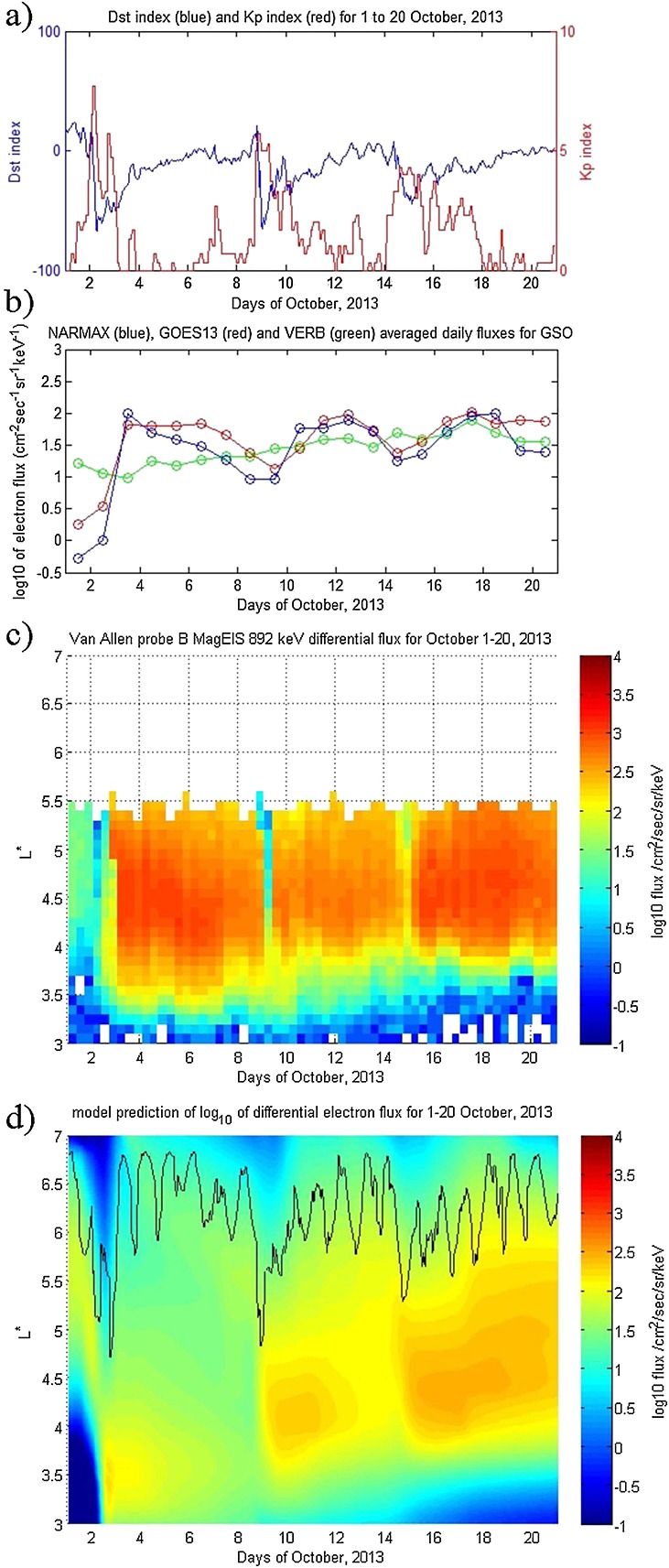
For 1–20 October 2013, (a) *Dst* and *Kp* indices, (b) differential flux for NARMAX, GOES-13, and VERB, (c) Van Allen probe B MagEIS data for 892 keV, and (d) VERB simulation of the outer radiation belt using the NARMAX-derived differential flux as the outer boundary. The black curve in Figure 3d represents geostationary orbit.

This period features three strong flux enhancement periods that can be seen on Figure 3: 2–9 October, 9–15 October, and 15–20 October. Sharp dropouts are evident between these time periods (Figure 3c) that can be characterized as main phase depletions from the *Dst* index on Figure 3a. The three events here appear to be caused by strong injections from the outer boundary, captured by GOES-13 at GSO and predicted successfully by NARMAX (Figure 3b). Meanwhile, the profile of the *Kp* index is unusual in that it contains periods of very low geomagnetic activity, then rapid rise to high indices (as high as *Kp* = 7 on 2 October) and shortly afterward, a drop back to very low activity.

Under these conditions, the simulation results begin to deviate from the observations. A small enhancement (1–2 October) from above L^*^≈ 5.5 down to L^*^ = 4.5 is observed on both the model and observations, followed by a sharp dropout on 2 October. However, the enhancement afterward (2–9 October), although visible on the model, is over an order of magnitude smaller than in observations. There are several possible reasons for this discrepancy. First, NARMAX underestimates GSO flux on 2–3 October (as is evident by the second blue and red data points on Figure 3b). As a result, flux at the outer boundary is underestimated, and this carries through into lower L shells. Second, local acceleration and radial diffusion rapidly drop off in the simulation after 3 September because they are parameterized by the *Kp* index, which drops to near zero. Since the convected population is underestimated, and little local acceleration occurs, the result is an underestimate in fluxes across the whole outer belt for that time period. The peak in fluxes in the simulation around 2–5 October is also around L^*^ = 3.5 while the observations suggest that it is around L^*^=4 − 4.5. The peak in flux in the simulation is most likely as a result of electrons adiabatically accelerated because of high geomagnetic activity. The observations do show an enhancement inward of L^*^ = 4, but electron fluxes below L^*^ = 3.5 fall off rapidly, likely due to scattering by plasmaspheric hiss. It is possible that the model underestimates the effect of plasmaspheric hiss during this time period, perhaps due to the unusual *Kp* index dynamics.

The other two enhancements (9–15 October and 15–20 October) are predicted more accurately, with fluxes peaking around L^*^4 − 4.5, consistently with observations. Flux drops around 8–9 October and 14–15 October are also seen in the model and observations. In both enhancements, the magnitude of fluxes is again underestimated by the model. Meanwhile, the effect of hiss scattering below L^*^ = 4 appears to be underestimated by the model as well, resulting in a less clear outer belt edge than observed by MagEIS. Since the effect of all waves appears to be underestimated, it is possible to suggest that the source of the discrepancy is the wave model because of very rapid changes in *Kp* index. Any inaccuracy with outer boundary flux will also carry through into local acceleration, since the outer boundary provides the seed and source population for the outer belt.

### 5.4 Geostationary Orbit

**Figure 4:**
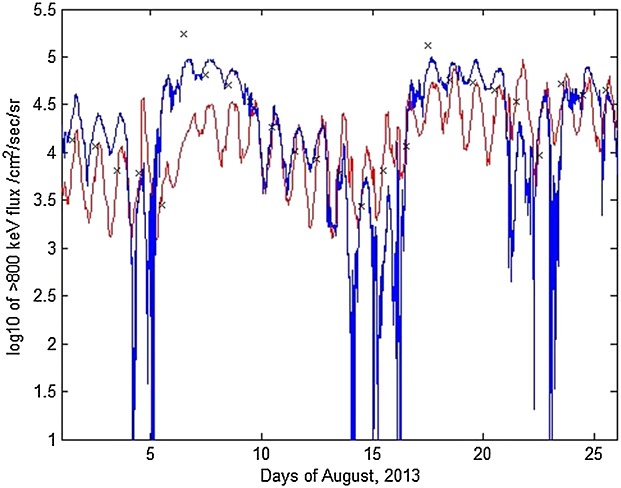
For 1–25 August 2013, > 800 keV flux at geosynchronous orbit (blue) versus model (red). Black crosses mark scaled NARMAX predictions for the days shown.

**Figure 5:**
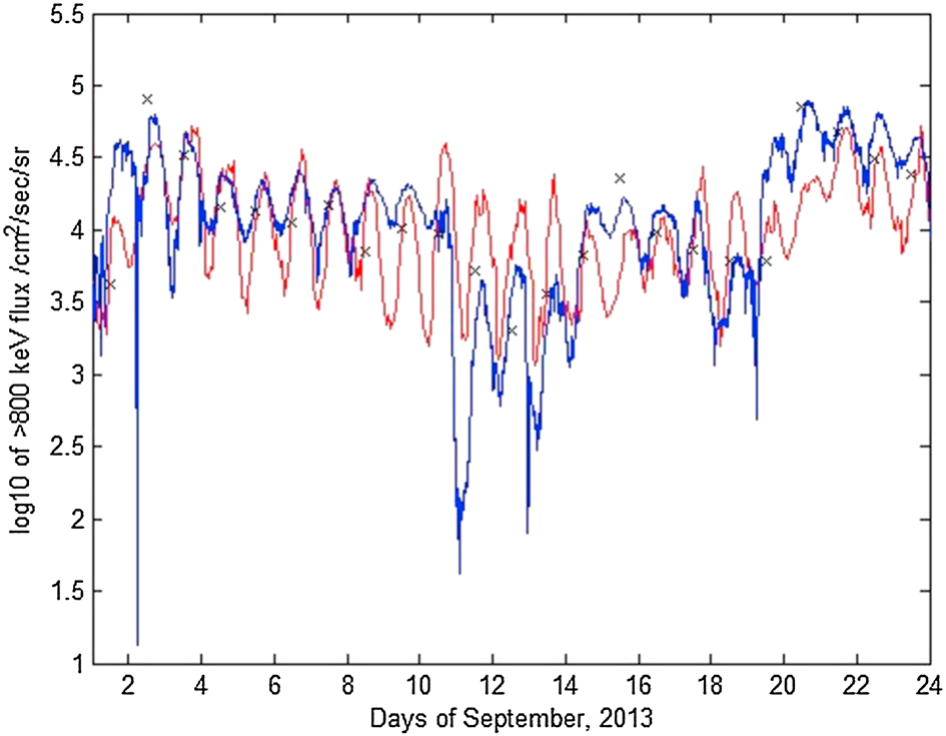
For 1–23 September 2013, > 800 keV flux at geosynchronous orbit (blue) versus model (red). Black crosses mark scaled NARMAX predictions for the days shown.

Figure 4 shows GOES-13 fluxes for the > 800 keV channel compared with fluxes predicted by VNC. Differential fluxes were obtained for two energies—891 keV and 1 MeV—and used to calculate > 800 keV integral fluxes assuming exponential energy distributions. It can be seen that, absent some underestimations at periods of rapid flux decrease, the model follows the data reasonably well. The same analysis was performed on the 1–23 September 2013 time interval, with the results visible on Figure 5. As noted above, the sinusoidal flux variation is a diurnal effect due to the spacecraft traversing different L^*^ regions at different magnetic local times. Black crosses on the Figures 4 and 5 show NARMAX predictions for those time periods. It can be seen that NARMAX predicts variations quite well at geostationary orbit and can be successfully used for outer boundary information as an input to the VERB system.

**Figure 6:**
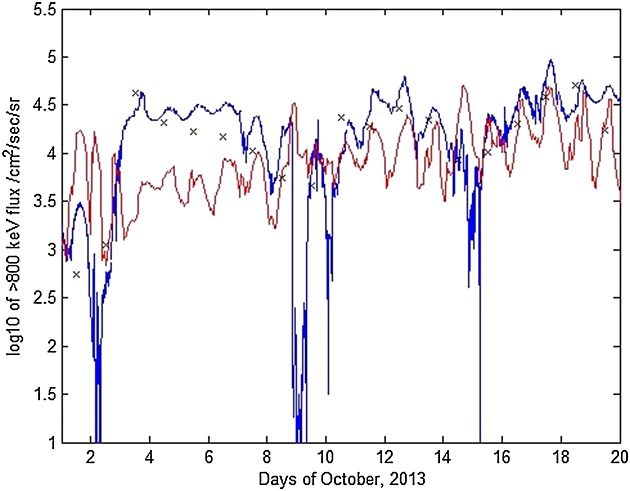
For 1–20 October 2013, > 800 keV flux at geosynchronous orbit (blue) versus model (red). Black crosses mark scaled NARMAX predictions for the days shown.

Figure 6 shows geosynchronous orbit data for 1–20 October 2013. Under the extreme geomagnetic conditions presented, the model (red) differ from the data (blue). In particular, the model estimate of the first enhancement around 3–4 October is over an order of magnitude lower than that observed. However, NARMAX predicts the enhancement effectively, as can be seen by the third black cross for 3 October. This suggests that the increased flux simply does not propagate from the outer model boundary to lower L^*^ values. The reason for this could be the rapid drop in all diffusion coefficients as the *Kp* index suddenly drops on 3 October. This would mean that any increases in outer boundary flux do not diffuse below the outer boundary, and there are few particles inside the belt to accelerate. Further, as mentioned above, local acceleration and pitch angle scattering would also be reduced under low *Kp* indices. Finally, the choice of magnetic field model can play a part since solar wind data and *Dst* index are not explicitly included in the T89 model.

The other two enhancements are captured more accurately, though it can be seen that model fluxes on 9–15 October and 15–20 October are still somewhat lower than those observed on GOES-13. Flux depletions occur too rapidly (on the scale of a few hours) for the model to react to them.

## 6. Caveats for Real-Time Forecasting

### 6.1 NARMAX as an Outer Boundary Driver

This study presents a first attempt to use predictions for GOES-13 data to drive a radiation belt diffusion code. It is believed that the NARMAX segment of the model would benefit from a higher time resolution. Rapid changes in GSO flux should then be captured sooner. In an environment of rapidly changing geomagnetic activity, a situation can arise where a rapid flux buildup or drop is only registered by the model 24 h later, by which point the geomagnetic indices driving the wave model may have changed completely. This propagates the error from the outer boundary into the heart of the belt. Further, at high geomagnetic activity the location of GSO in L^*^ may drop below L^*^ = 6.2, meaning the flux transfer operation may no longer be valid. One solution is to set the outer boundary lower. However, this may affect GSO prediction capacity, as a low boundary can take GSO out of the simulation domain.

High-energy channels (> 800 keV and > 2 MeV) are used for outer boundary flux determination. Meanwhile, it is believed that convected lower energy electrons form the source and seed populations for the radiation belt. A lower energy channel may thus give better predictions, in particular with a higher time resolution that will allow to isolate fluxes from nightside MLT sectors only. Work to this end is currently ongoing.

### 6.2 Magnetopause Shadowing

Magnetopause shadowing is a phenomenon caused by a pressure pulse from the solar wind, for example, a fast CME, compressing the magnetosphere. If the boundary of the magnetosphere is near geosynchronous orbit, the drifting radiation belt electrons impact the magnetopause and are lost from the system to the solar wind. This causes a rapid drop in particle counts [e.g., *Shprits et al.*, 2006a,2006b; *Horne et al.*, 2013], affecting all energies [e.g., *Bortnik et al.*, 2006] and potentially accounting for over 90% of electron loss outward of L^*^ = 5 [*Yu et al.*, 2013]. Since the pressure pulse in the solar wind can only be detected at L1 and takes about 40 min to reach the Earth, it is hard to predict for such an event several hours in advance. However, since dropouts cause depletions, rather than buildups, of relativistic electrons, it will lead to an over-prediction of flux levels and should not pose any real risk. Improving the coupling between the model and the solar wind together with a model for magnetopause location [*Shue et al.*, 1997] is a subject of current work.

### 6.3 *Kp* Index

The *Kp* index is used to drive both the VERB model (where it influences the location and effect of the local diffusion region) and also the T89 model for calculating the magnetic shell and constructing the diurnal variation dynamic. It is well known [e.g., *Roederer*, 1970] that MLT location accounts for most of the variation of energetic electron flux, and it is seen again in the observations above. Thus having an accurate measure of *Kp* index is crucial for the success of the prediction. To move toward forecasting, a model that predicts *Kp* index should be employed, such as the Wing *Kp* forecast model [*Wing et al.*, 2005]. Research to this end is currently ongoing.

### 6.4 Computing Time Considerations

As outlined in *Subbotin et al.* [2010], including the full Fokker-Planck diffusion equation involves including mixed energy and pitch angle diffusion coefficients. This can place computational strain on the model and slow down the simulation. One effect of including mixed diffusion terms is an effective inhibition of local acceleration; the peak in PSD associated with local acceleration forms later and is not as large as when mixed diffusion terms are omitted. Changes to the dynamics of the source region in turn affect simulated fluxes at geostationary orbit, and it was found that the VERB-NARMAX model performance was improved by inclusion of the mixed terms. All the above simulations were performed with mixed diffusion turned on. In practical terms, if the aim is to create a system that would generate forecasts every hour, the system has to physically have time to run all the necessary calculations. This can be done by assigning the simulations as a series of batch jobs to a supercomputer or by running the simulation with a reduced grid size or for smaller time periods. Omission of the mixed diffusion terms has been found to produce a satisfactory fit to observations, although with reduced accuracy.

## 7. Summary

A model fusing the data-driven NARMAX and physics-driven VERB codes has been developed and tested. The model combines the accuracy of the data-driven approach with the predictive range and resolution of physical methodology. The coupled simulation was tested on three separate time periods, simulating 68 days in total. The aim was to test the long-term performance of the model at predicting energetic electron fluxes at and inside of geostationary orbit in conditions typically encountered in the magnetosphere. The time periods studied contain all typical features of radiation belt dynamics: SSCs, dropouts, refilling, and rapid changes in geomagnetic indices. The combined system was found to reasonably accurately model fluxes during quiet and disturbed conditions, capturing all the essential dynamics. Sources of discrepancies, as well as caveats for the use of the model in real-time radiation belt forecasting, have been outlined in the manuscript.

These simulations show that the dynamic evolution of the outer regions of the radiation belts presents a strong driver of the dynamics of the radiation belts. *Kim et al.* [2012] further showed that losses to the magnetopause can extend to L shells much lower than VERB typical outer boundary and may explain overestimation of fluxes during dropout events. Accurate simulations of the dropouts will include accounting for the MLT-dependent loss to the magnetopause and accurate simulation of adiabatic effects during storms [*Kim et al.*, 2010; *Saito et al.*, 2010; *Su et al.*, 2011; *Kim and Chan*, 1997]. The model has been found to slightly overestimate energetic electron fluxes, especially during periods of rapid radiation belt depletion, but simulates radiation belt rebuilding with good accuracy. Data from the Van Allen probes showed good agreement and allowed to validate the system for the entire outer radiation belt region.

Our simulations also confirm suggestions of *Kim et al.* [2012] that losses to the magnetopause can extend to the heart of the inner belt and cannot be modeled simply by the variations of the outer boundary. Future work should include MLT dependent simulations that account for convection, adiabatic effects and MLT- and pitch-angle-dependent loss to the magnetopause.

The study shows that high-energy electron fluxes near the outer boundary can be used to predict radiation belt dynamics. Since convection mainly brings low-energy particles to the radiation belts, this is somewhat surprising, particularly considering the low time resolution of NARMAX. In essence, NARMAX in its current form captures all high-energy particles at GSO, including those locally accelerated inside the belt. An intriguing question emerges—what proportion of high-energy particles observed at GSO are the high-energy tail of an injected population, and what proportion are locally accelerated electrons that have then radially diffused outward to GSO? A comparative study of the effectiveness of low-energy and high-energy channels from GSO satellites would shed light on this matter.

## Key Points

- Physics-and-data coupled code developed
- Tested for several weeks of representative storms
- Reproduces observations, discrepancies discussed
